# Cisplatin Nephrotoxicity Is Critically Mediated by the Availability of BECLIN1

**DOI:** 10.3390/ijms25052560

**Published:** 2024-02-22

**Authors:** Tillmann Bork, Camila Hernando-Erhard, Wei Liang, Zhejia Tian, Kosuke Yamahara, Tobias B. Huber

**Affiliations:** 1 Department of Medicine IV, Faculty of Medicine, University of Freiburg, 79106 Freiburg, Germany; tillmann.bork@uniklinik-freiburg.de (T.B.);; 2 Division of Nephrology, Renmin Hospital of Wuhan University, Wuhan 430064, China; 3 Department of Nephrology and Hypertension, Hannover Medical School, 30625 Hannover, Germany; tian.zhejia@mh-hannover.de; 4 Department of Medicine, Shiga University of Medical Science, Tsukinowa-cho, Otsu 520-2192, Shiga, Japan; 5 III Department of Medicine, University Medical Center Hamburg-Eppendorf, 20246 Hamburg, Germany

**Keywords:** AKI, BECLIN1, autophagy, apoptosis, cisplatin, ER stress

## Abstract

Cisplatin nephrotoxicity is a critical limitation of solid cancer treatment. Until now, the complex interplay of various pathophysiological mechanisms leading to proximal tubular cell apoptosis after cisplatin exposure has not been fully understood. In our study, we assessed the role of the autophagy-related protein BECLIN1 (ATG6) in cisplatin-induced acute renal injury (AKI)—a candidate protein involved in autophagy and with putative impact on apoptosis by harboring a B-cell lymphoma 2 (BCL2) interaction site of unknown significance. By using mice with heterozygous deletion of *Becn1*, we demonstrate that reduced intracellular content of BECLIN1 does not impact renal function or autophagy within 12 months. However, these mice were significantly sensitized towards cisplatin-induced AKI, and by using *Becn1^+/−^;Sglt2-Cre;Tomato/EGFP* mice with subsequent primary cell analysis, we confirmed that nephrotoxicity depends on proximal tubular BECLIN1 content. Mechanistically, BECLIN1 did not impact autophagy or primarily the apoptotic pathway. In fact, a lack of BECLIN1 sensitized mice towards cisplatin-induced ER stress. Accordingly, the ER stress inhibitor tauroursodeoxycholic acid (TUDCA) blunted cisplatin-induced cell death in *Becn1* heterozygosity. In conclusion, our data first highlight a novel role of BECLIN1 in protecting against cellular ER stress independent from autophagy. These novel findings open new therapeutic avenues to intervene in this important intracellular stress response pathway with a promising impact on future AKI management.

## 1. Introduction

Acute Kidney Injury (AKI) is a frequent syndrome defined as an abrupt decrease in kidney function based on an increase in serum creatinine and a reduction in urine output [1,2,3]. AKI is associated with poor clinical outcome as seen in numerous clinical studies reporting an increased risk for chronic kidney disease (CKD) and end-stage renal disease (ESRD) following de novo AKI [4,5]. AKI frequently occurs in the presence of sepsis, during post-resuscitation care or after exposure to nephrotoxic drugs. A candidate drug with high nephrotoxic potential is cisplatin.

Cisplatin (cis-diamminedichloroplatinum II, CDDP) is a first-generation platinum derivative highly effective in the treatment of various types of cancer such as testicular, esophageal, pancreatic, stomach and lung cancer [6,7,8,9,10]. However, nephrotoxic side-effects of cisplatin treatment are common and occur in approximately 28% to 36% of patients treated with cisplatin [11,12]. Toxic effects are not limited to transient AKI but are also associated with CKD progression, thereby leading to increased co-morbidity [13].

Cisplatin accumulates in proximal tubular cells mainly due to excessive cellular uptake, mainly via proximal tubule organic cation transporter 2 (OCT2) [14,15]. The cellular mechanisms of cisplatin toxicity include (among others) a reduced expression of glucose, amino acid, magnesium and water transporters [16,17]; activation of autophagy; activation of the mitogen-activated protein kinase (MAPK) signaling pathways; DNA damage; ER stress; and mitochondrial dysfunction [18,19,20]. These mechanisms finally lead to proximal tubular cell death by activating the apoptotic pathway. The crucial role of apoptosis as the final effector of cisplatin toxicity has been proven in mice with genetic deletion of the proapoptotic BAX protein [21]. These mice became resistant to cisplatin exposure. Furthermore, anti-CASPASE12 antibodies were able to attenuate cisplatin-induced cell death, indicating a major role of the intrinsic apoptotic pathway—activated by cisplatin-induced ER stress [22].

The well-described role of apoptosis in cisplatin nephropathy allows for in vivo studies of other proteins with putative involvement in apoptotic pathways. A candidate protein potentially involved in the apoptosis cascade is the autophagy-related (ATG) protein BECLIN1. BECLIN1 harbors three domains promoting protein–protein interaction: The evolutionarily conserved domain (ECD) promotes class III phosphatidylinositol 3’-kinase (PI3K)/VPS34 binding and thereby autophagy, the coiled coli domain (CCD) allows for interaction with other ATG proteins, namely UVRAG and RUBICON. The BH3 domain interacts with B-cell lymphoma 2 protein (BCL-2) and B-cell lymphoma-extra large protein (BCL-xL), both modulating the apoptotic pathway [23,24,25]. However, until now, direct evidence for an involvement of BECLIN1 in apoptosis has been missing.

By using mice with heterozygous deletion of the *Beclin1* gene (*Becn1*^+/−^), we aimed to clarify a direct involvement of BECLIN1 in apoptosis induced by cisplatin exposure.

## 2. Results

### 2.1. Heterozygous Disruption of Becn1 Promotes Cisplatin-Induced Nephropathy In Vivo

**Figure 1:**
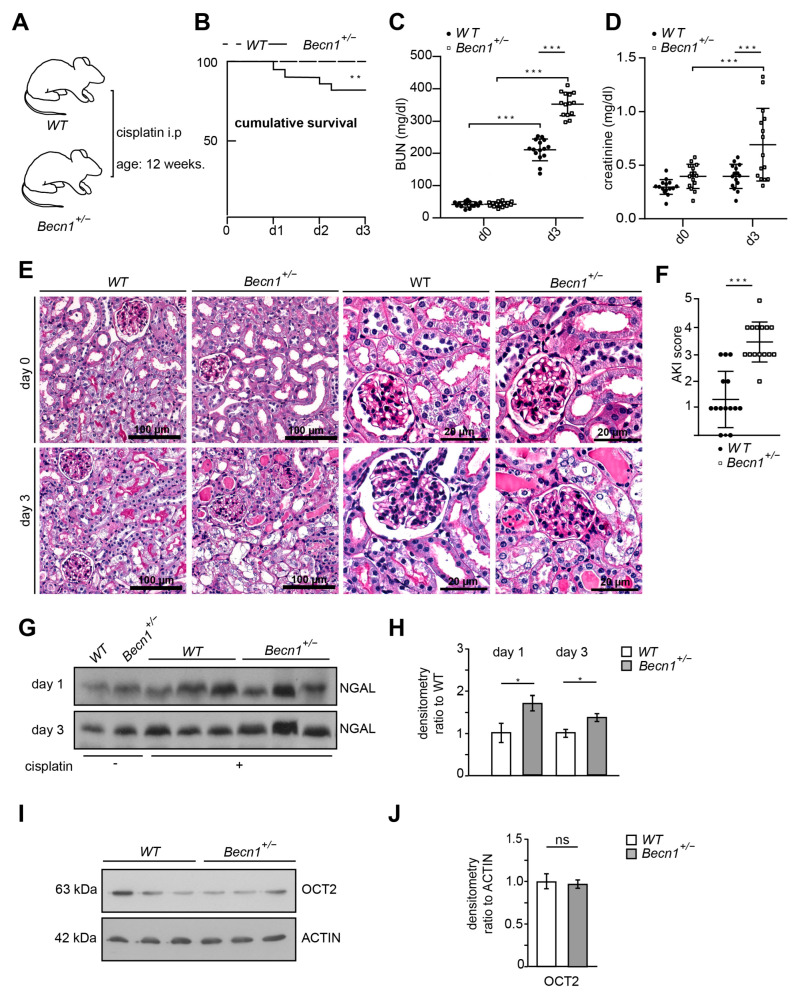
*Becn1^+/−^* sensitizes towards cisplatin nephropathy: (**A**) Schematic of the study protocol. (**B**) Cumulative survival of WT and *Becn1^+/−^* mice after cisplatin exposure (*n* = 15 each group, ** ≤ 0.01). (**C**) BUN of WT and *Becn1^+/−^* mice after cisplatin exposure (*n* = 15 each group, dose: 20 mg/kg, *** ≤ 0.001). (**D**) Serum creatinine of WT and *Bec1^+/−^* mice after cisplatin exposure (*n* = 15, *** ≤ 0.001). (**E**) Representative kidney sections stained with hematoxylin–eosin solution obtained from WT and *Becn1^+/−^* mice after cisplatin exposure at the time point as indicated. (**F**) AKI score obtained from WT and *Becn1^+/−^* mice after cisplatin exposure (*n* = 15, *** ≤ 0.001). (**G**) Western blot showing the abundance of urinary NGAL from WT and *Becn1^+/−^* mice after cisplatin exposure. (**H**) Densitometry for the abundance of urinary NGAL from WT and *Becn1^+/−^* mice after cisplatin exposure (*n* = 3; * ≤ 0.05). (**I**) Western blot showing the abundance of OCT2 in kidney lysates obtained from WT and *Becn1^+/−^* mice. (**J**) Densitometry for the abundance of OCT2 in kidney lysates obtained from WT and *Becn1^+/−^* mice (*n* = 3, ns = non significant).

Since complete knock-out of *Becn1* leads to severe cellular phenotypes in vitro and in vivo, we studied mice with constitutive heterozygous disruption of *Becn1*. These mice showed reduced levels of BECLIN1 (Figure S1A,B); however, their lifespan, renal function and proteinuria were not affected (Figure S1C–F). Kidney sections obtained from *Becn1*^+/−^ and WT mice showed no difference up to 12 months of age—neither in the glomerular nor in the tubular compartments (Figure S1G–I). Given that heterozygous disruption of *Becn1* did not display any renal phenotype per se within the first 12 months, we exposed 12-week-old male WT and *Becn1*^+/−^ mice to cisplatin (Figure 1A). In this regimen, WT mice were hardly affected, which clearly delineates a potential BECLIN1-dependent sensitization to cisplatin nephropathy. In fact, *Becn1*^+/−^ mice showed a clear increase in mortality and a significantly worse renal outcome indicated by increased levels of blood urea nitrogen and serum creatinine (Figure 1B–D). Histopathological analysis of kidney sections revealed massive renal deterioration in *Becn1*^+/−^ mice with tubular brush boarder loss, apoptosis and tubular cast formation (Figure 1E). Quantification based on histopathological AKI criteria revealed significantly increased renal deterioration in *Becn1*^+/−^ mice (Figure 1F). Accordingly, urinary neutrophil gelatinase-associated lipocalin (NGAL) excretion was significantly increased in these mice, indicating more severe AKI manifestation if renal BECLIN1 abundance is reduced (Figure 1G,H). Since BECLIN1 impacts anterograde Golgi trafficking with a potential subsequent impact on the abundance of membrane-bound transporters, OCT2 levels have been assessed [26]. Comparable expression levels of OCT2 suggest no differences in the tubular uptake capacity of cisplatin in *Becn1*^+/−^ or WT mice (Figure 1I,J).

### 2.2. Heterozygous Disruption of Becn1 Sensitizes to Apoptosis In Vivo and In Vitro

**Figure 2:**
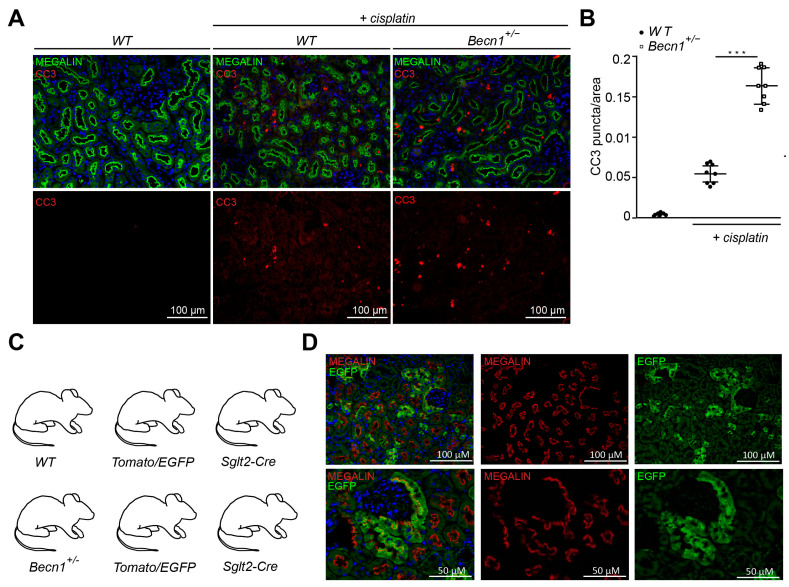

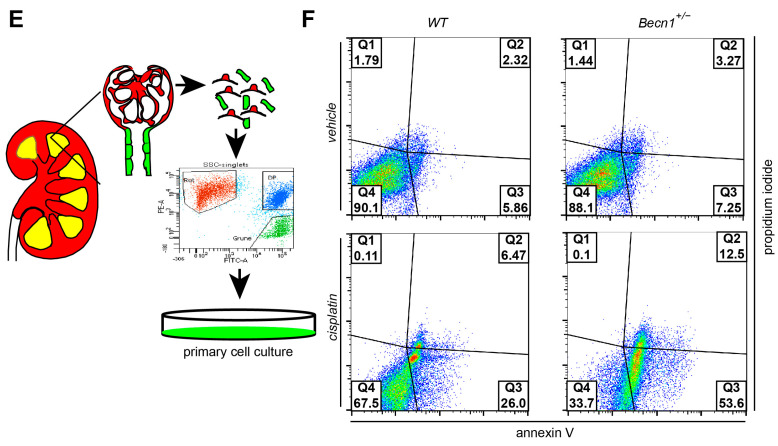
*Becn1^+/−^* sensitizes mice towards tubular cell apoptosis cisplatin nephropathy: (**A**) Representative immunofluorescence staining of kidney section obtained from WT and *Becn1^+/−^* mice with and without cisplatin exposure for MEGALIN and cleaved CASPASE3. (**B**) Quantification of cleaved CASPASE3 signal in kidney section obtained from WT and *Becn1^+/−^* mice with and without cisplatin exposure (*n* = 8, *** ≤ 0.001). (**C**) WT and *Becn1^+/−^* mice were crossed to *Sglt2-Cre* and *Tomato/EGFP* mice to obtain green fluorescent proximal tubular cells. (**D**) Representative immunofluorescence staining of kidney section obtained from *Becn1*^+/−^;*Tomato/EGFP*;*Sglt2-Cre* mice and corresponding WT controls for MEGALIN and endogenous EGFP. (**E**) Schematic for the generation of primary proximal tubular cells from *Becn1*^+/−^;*Tomato/EGFP*;*Sglt2-Cre* mice and corresponding WT controls. (**F**) Flow cytometric analysis result of annexin and propidium iodide staining for the assessment of apoptosis in primary proximal tubular cells obtained from *Becn1*^+/−^;*Tomato/EGFP*;*Sglt2-Cre* mice and corresponding WT controls with and without cisplatin exposure.

To gain further insights into the mechanism of renal deterioration in cisplatin-exposed *Becn1*^+/−^ mice, apoptotic markers were assessed. Immunostaining for cleaved CASPASE3 revealed a high activity of the apoptotic pathway in *Becn1*^+/−^ mice after cisplatin exposure (Figure 2A,B). For further analysis, we crossed *Becn1*^+/−^ mice with *Tomato/EGFP* reporter mice and *Sglt2-Cre* mice (Figure 2C). We thereby generated mice with green fluorescent proximal tubular cells (Figure 2D). This genetic cell-specific labeling allowed for primary tubular cell culture after kidney dissection, digestion and sieving with subsequent fluorescence-activated cell sorting (FACS) (Figure 2E). We thereby generated WT and *Becn1*^+/−^ primary proximal tubular cells and exposed these cells to cisplatin. FACS-based analysis of apoptosis was performed by using annexin V and propidium iodide (PI) staining. According to our in vivo data, ex vivo-exposed primary proximal tubular cells also showed a significant increase in apoptosis after cisplatin exposure if the amount of BECLIN1 was reduced (Figure 2F).

Since BECLIN1 harbors a BH3 domain, direct involvement in apoptosis as known from other BH3 proteins seemed to be possible explanation of our finding. For further analysis, knock-down of *Becn1* was performed by using siRNA in human embryonic kidney (HEK) cells, reducing intracellular BECLIN1 content (Figure S2A). Subsequently, FACS-based analysis of apoptosis was performed by using annexin V and propidium iodide labeling after cisplatin exposure (Figure S2B). Knock-down of *Becn1* resulted in a higher proportion of annexin V-positive cells (86.2% vs. 44.3% in controls) and annexin V/PI double-positive cells (11.2% vs. 10.8% in controls). These results correspond with our in vivo findings and suggest an anti-apoptotic effect of BECLIN1. To further test this hypothesis, overexpression of Beclin1 was performed with a massive increase in BECLIN1 abundance (Figure S2C). FACS-based analysis of apoptosis after cisplatin exposure, however, did not show reduced apoptosis but revealed a slightly higher proportion of annexin V-positive cells with BECLIN1 overexpression (35.8% vs. 26.3% in controls) and annexin V/PI double-positive cells (14.6% vs. 11.2% in controls) (Figure S2D). In summary, a direct pro- or anti-apoptotic effect as known from other BH3 proteins could not be detected.

### 2.3. Cisplatin Induces Autophagy Independent from BECLIN1 Abundance In Vivo

**Figure 3:**
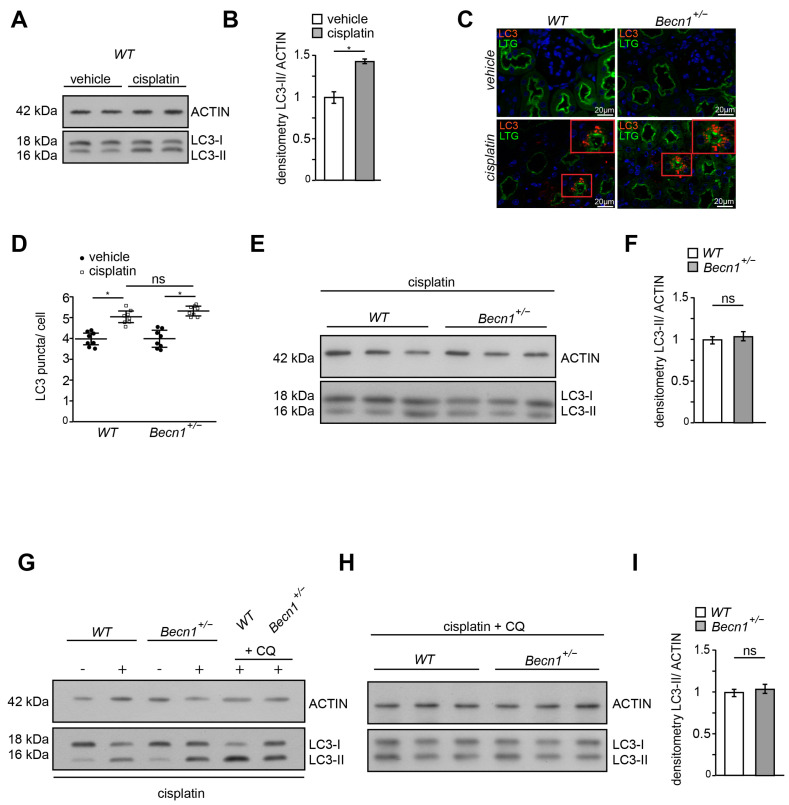
Cisplatin induces autophagy independent from BECLIN1 abundance in vivo: (**A**) Western blot for the abundance of LC3 and β-ACTIN in lysates obtained from kidney of vehicle- or cisplatin-exposed mice (after 3 days, dose: 20 mg/kg). (**B**) Densitometry for the abundance of LC3-II in lysates obtained from kidney of vehicle- or cisplatin-exposed mice (*n* = 3, * ≤ 0.05). (**C**) Immunofluorescence staining for LTG and LC3 in kidney section obtained from WT and *Becn1^+/−^* mice with and without cisplatin exposure. (**D**) Quantification of LC3 signal in kidney section obtained from WT and *Becn1^+/−^* mice with and without cisplatin exposure (*n* = 8, * ≤ 0.05, ns = non significant). (**E**) Western blot for the abundance of LC3 and β-ACTIN in lysates obtained from kidneys of vehicle- or cisplatin-exposed WT and *Becn1^+/−^* mice (short exposure for LC3). (**F**) Densitometry for the abundance of LC3-II in lysates obtained from kidneys of cisplatin-exposed WT and *Becn1^+/−^* mice (*n* = 3, ns = non significant). (**G**) Western blot for the abundance of LC3 and ß-ACTIN in lysates obtained from primary proximal tubular *Becn1^+/−^* and *WT* cells after exposure to cisplatin (200 µmol/L, 12 h) with and without chloroquine (100 µmol/L, 12 h). (**H**) Western blot for the abundance of LC3 and β-ACTIN in lysates obtained from primary proximal tubular *Becn1^+/−^* and *WT* cells after exposure to cisplatin (200 µmol/L, 12 h) with chloroquine (100 µmol/L, 12 h). (**I**) Densitometry for the abundance of LC3-II in lysates obtained from primary proximal tubular *Becn1^+/−^* and *WT* cells after exposure to cisplatin (*n* = 3, ns = non significant).

Since BECLIN1 is a key ATG involved in autophagy and autophagy has been shown to prevent cisplatin-induced renal deterioration, we assessed LC3 lipidation in renal lysates obtained from *WT* mice with and without cisplatin exposure. As indicated by an increased abundance of lipidated LC3 (namely LC3-II), autophagy was activated by cisplatin (Figure 3A,B). Comparing the *WT* and *Becn1*^+/−^ mice after cisplatin exposure, increased autophagosome formation was observed in both genotypes by the assessment of LC3 puncta in immunofluorescence (Figure 3C). Reduced abundance of BECLIN1 did not impact the number of formed autophagosomes in proximal tubular cells (Figure 3D). Correspondingly, the abundance of LC3-II in kidney lysates revealed no difference in autophagy between *WT* and *Becn1*^+/−^ mice (Figure 3E,F). In primary proximal tubular cells obtained from *Becn1*^+/−^;*Tomato/EGFP*;*Sglt2-Cre* and the corresponding WT controls, cisplatin activated autophagy and autophagic flux as well (Figure 3G). The assessment of autophagic flux by using lysosomal inhibition by chloroquine showed no difference in *WT* and *Becn1*^+/−^ primary proximal tubular cells (Figure 3H–I). These data confirm that differences in BECLIN1 abundance did not impact autophagy activation following cisplatin exposure, thereby suggesting another role other than autophagy for BECLIN1 in cisplatin-induced nephropathy.

### 2.4. Cisplatin-Induced ER Stress Depends on BECLIN1 and Activates the Apoptotic Pathway

**Figure 4:**
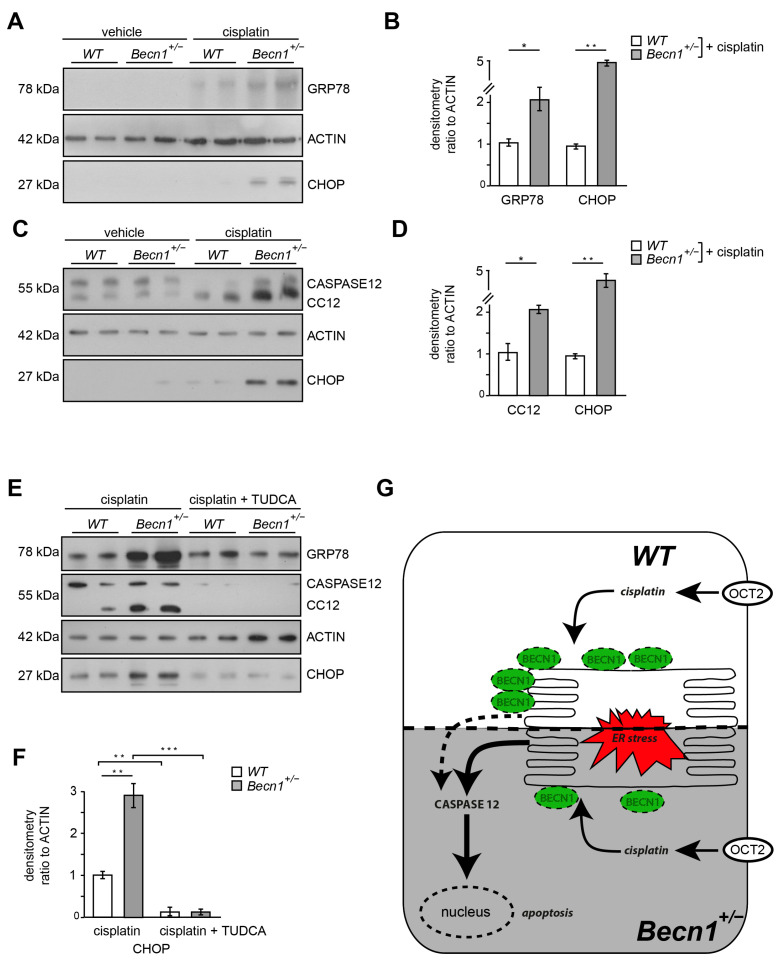
Cisplatin-induced ER stress depends on BECLIN1 and activates the apoptotic pathway: (**A**) Western blot for the abundance of GRP78, CHOP and β-ACTIN in lysates obtained from kidneys of vehicle- or cisplatin-exposed *Becn1^+/−^* and *WT* mice (after 3 days, dose: 20 mg/kg). (**B**) Densitometry for the abundance of GRP78 and CHOP in lysates obtained from kidneys of vehicle- or cisplatin-exposed *Becn1^+/−^* and *WT* mice (*n* = 3, * ≤ 0.05, ** ≤ 0.01). (**C**) Western blot for the abundance of CASPASE12, CHOP and β-ACTIN in lysates obtained from primary proximal tubular *Becn1^+/−^* and *WT* cells after exposure to cisplatin (200 µmol/L, 12 h). (**D**) Densitometry for the abundance of CASPASE12 and CHOP in lysates obtained from primary proximal tubular *Becn1^+/−^* and *WT* cells after exposure to cisplatin (*n* = 3, * ≤ 0.05, ** ≤ 0.01). (**E**) Western blot for the abundance of GRP78, cleaved CASPASE12, CHOP and β-ACTIN in lysates obtained from primary proximal tubular *Becn1^+/−^* and *WT* cells after exposure to cisplatin with and without TUDCA (100 μM, 12 h). (**F**) Densitometry for the abundance of CHOP in lysates obtained from primary proximal tubular *Becn1^+/−^* and *WT* cells after exposure to cisplatin with and without TUDCA (*n* = 3, ** ≤ 0.01, *** ≤ 0.001). (**G**) Schematic for ER protection by BECLIN1 in proximal tubular cells after cisplatin exposure.

In WT mice, BECLIN1 levels increased after cisplatin exposure (Figure S3A). In *Becn1*^+/−^ mice, however, this upregulation after cisplatin exposure was blunted (Figure S3B). These data suggest a role of BECLIN1 in cellular stress response. Since cisplatin increases ER stress and BECLIN1 has been shown to localize to ER, we assessed markers of ER stress in kidney lysates of *Becn1*^+/−^ and WT mice after cisplatin exposure [27,28]. ER stress markers GRP78 and CHOP massively increased in *Becn1*^+/−^ mice compared to WT (Figure 4A,B). In vitro, primary proximal tubular cells obtained from WT mice showed a moderate increase in ER stress after cisplatin exposure, whereas in *Becn1*^+/−^ primary proximal tubular cells ER stress was massively increased (Figure 4C–E). Accordingly, CASPASE12 cleavage was strongly increased in *Becn1*^+/−^ proximal tubular cells, indicating high activity of the intrinsic apoptotic pathway. To further clarify the contribution of ER stress as the key mechanism in the observed phenotype in heterozygous disruption of *Beclin1,* pharmacological inhibition of ER stress was performed by using tauroursodeoxycholic acid (TUDCA). Concomitant treatment with the ER stress inhibitor TUDCA significantly reduced ER stress in *Becn1*^+/−^ proximal tubular cells as indicated by reduced GRP78 and CHOP abundance (Figure 4E,F and Figure S3C). Furthermore, cleavage of CASPASE12 was blunted, indicating less activation of the apoptotic pathway if ER stress was reduced. In summary, intracellular BECLIN1 content critically impacts cisplatin toxicity in proximal tubular cells by affecting ER susceptibility in an abundance-dependent manner (Figure 4G).

## 3. Discussion

BECLIN1 has been described as a multifunctional protein involved in autophagy, endocytosis and vesicle trafficking [29,30,31]. Furthermore, the BH3 domain of BECLIN1 suggest an involvement of this protein in the apoptotic cascade as known from other BH3-containing proteins such as BCL2, BAX/BAD, PUMA, BIM or others [32,33]. To further investigate the putative role of BECLIN1 in apoptosis, we analyzed the effect of reduced BECLIN1 levels in vivo in cisplatin-induced nephropathy—a candidate model for proximal tubular cell apoptosis. Our data clearly indicate that heterozygous deletion of *Becn1* sensitized mice for cisplatin nephrotoxicity and subsequent nephropathy.

Since complete knock-out of *Becn1* results in cellular deterioration with subsequent cell death, we chose a mouse model with a constitutive heterozygous deletion of *Becn1^+/−^* to clarify its role in cisplatin nephropathy [26,34,35]. Heterozygous deletion leads to an increased frequency of spontaneous malignancies in mice older than 13 months [36]. Our detailed analysis, however, revealed no renal phenotype after up to 12 months, thereby excluding any severe pre-existing renal impairment. To exclude extra renal effects of conditional *Becn1* heterozygosity after cisplatin exposure, we confirmed our findings by using primary proximal tubular cells. Furthermore, these primary cell studies allowed further insights into mechanisms of cisplatin toxicity and the role of BECLIN1 in this context. Since BECLIN1 is crucial for autophagy initiation, impaired autophagy appeared as an obvious explanation for increased susceptibility in *Becn1*^+/−^ mice. Numerous studies demonstrated that impaired autophagy sensitized mice to renal cisplatin toxicity. Genetic approaches using conditional knock-out of *Atg5* or *Atg7* in tubular cells revealed worsened AKI following cisplatin exposure in vivo [20,37,38]. Accordingly, the activation of autophagy, e.g., by rapamycin, showed a protective effect against cisplatin-induced renal deterioration [39,40]. Mechanistically, the protective effect of autophagy might be related to its role in mitochondrial renewal since mitochondria are particularly targeted in cisplatin toxicity [41]. Our experiments, however, revealed the same levels of autophagy activation in *Becn1*^+/−^ mice and WT, respectively, after cisplatin exposure, indicating that reduced levels of intracellular BECLIN1 content were not critical for autophagy. These findings are in contrast to previous studies demonstrating impaired autophagy in *Becn1* knock-down [19,42,43]. These studies, however, present in vitro data based on transient knock-downs achieved by siRNA or shRNA technology with time-dependent efficiency and putative off-target effects. In our study, however, we provide another level of evidence by using a genetically stable in vivo model with subsequent comprehensive primary cell studies based on various transgenic mice. Since autophagy was not critically altered in our study, we checked for direct pro- or antiapoptotic effects of BECLIN1. As mentioned above, structurally, BECLIN1 harbors different domains for a complex interaction with proteins involved in the apoptotic cascade, namely, BCL2 or others, which makes a regulatory role for BECLIN1 in apoptosis conceivable. However, especially in regard to the interplay with BCL2, BECLIN1 did not change the anti-apoptotic impact of BCL2 [44]. In fact, BECLIN1 itself has been reported to be cleaved by caspases, thereby leading to altered autophagy [45]. In our study, direct assessment of apoptosis in cells with various intracellular BECLIN1 contents did not show a clear correlation, indicating that apoptosis occurred in consequence to another role of BECLIN1, in proximal tubular cell homeostasis, rather than primarily serving as a signaling hub for apoptosis or autophagy, respectively.

BECLIN1 is enriched in cytoplasmic structures, including mitochondria, Golgi apparatus and the ER [29]. Functionally, BECLIN1 has been shown to localize to the ER during autophagy induction, thereby driving autophagosome formation [28]. Numerous studies dealing with autophagy/UPR/ER stress interplay suggest that impaired autophagy leads to protein aggregate accumulation in the ER, promoting ER stress [46,47]. This has been particularly shown for *Becn1* deficiency in vivo. In zebrafish, *Becn1* deficiency disrupts autophagic flux in the liver, leading to protein aggregate accumulation in the ER with subsequent severe ER stress and apoptosis [48]. Our data, however, showed comparable levels of autophagosome formation in *Becn1*^+/−^ mice in vivo and in vitro complemented with autophagic flux assessment. Yet, we showed a clear sensitization towards ER stress independent from autophagy.

ER stress plays a key role in the pathogenesis of AKI as demonstrated in various experimental models as well as in human biopsy samples [49,50,51]. Accordingly, ER stress occurs as a prominent feature in cisplatin nephropathy [52,53,54]. However, how BECLIN1 impacts ER function in the context of cisplatin nephropathy remains elusive and has to be addressed in further studies. Since BECLIN1 plays a crucial role in membrane dynamics, impaired intracellular vesicle trafficking with subsequent alterations in organelle dynamics and functions might occur after cisplatin exposure if BECLIN1 content falls below a certain threshold. This hypothesis is supported by experimental evidence showing that BECLIN1 is involved in endocytosis and endosome-to-Golgi retrograde trafficking [55,56]. Also, anterograde trafficking relies on proper BECLIN1 function as shown by our group first describing a key role of BECLIN1 in secretory functions [26]. Sequestration of cisplatin into vesicles belonging to the secretory pathway as well as into lysosomes has been described as a mechanism for cisplatin resistance in tumor cells [57,58,59]. Our data raise the hypothesis that in proximal tubular cells proper vesicle formation is also essential for cellular stress resistance after cisplatin exposure. However, this has to be further elucidated in experimental studies.

Our study shows certain limitations. Technically, more extensive control data such as CASPASE3 assessment in *Becn1^+/−^* mice would be desirable. Furthermore, previous studies showed that other pathways of cell death such as necroptosis and ferroptosis play an important role in cisplatin nephropathy and these pathways were not in the scope of our study [60,61,62,63]. Also, inflammatory pathways with various mediators including tumor necrosis factor alpha (TNFα), toll-like receptor (TLR) signaling and chemokine (C-X-C motif) ligand (CXCL) 16 and the CXCL1-CXCR 2 axis have been shown to contribute to renal deterioration in cisplatin nephropathy [54,64,65,66,67,68]. Investigations of these pathways in the context of reduced BECLIN1 abundance in *Becn1*^+/−^ would be interesting and should be addressed in further studies since these aspects are beyond the scope of our current manuscript.

In conclusion, in our study, BECLIN1 emerged as a crucial regulator for ER stress response—a key pathogenic mechanism in AKI. Based on our various transgenic mouse models and comprehensive primary cell studies, we first report a critical role for proximal tubular BECLIN1 abundance in cisplatin nephropathy *independent* from autophagy. In the past, BECLIN1-derived peptides based on autophagy-inducing motifs of BECLIN1 have been successfully tested in preclinical studies to cure or prevent infectious diseases [69,70]. Our data highlight novel properties of BECLIN1 independent from autophagy. Our study thereby opens avenues for the development of other BECLIN1-derived compounds specifically targeting ER. Thereby, BECLIN1-derived peptides might bear high clinical potential in the prophylaxis of cisplatin nephropathy, allowing the dose-limiting renal toxicity of this potent anticancer drug to be overcome.

## 4. Materials and Methods

### 4.1. Mice

B6.129X1-*Becn1*^tm1Blev^/J (*Becn1^+/−^*) were generated by Qu et al. and purchased for our studies from JAX (stock #:018429) [36]. To assess the impact of *Becn1* heterozygosity on renal structure and function, a cohort of 30 *Becn1^+/−^* and 30 corresponding WT littermates were analyzed and 10 mice of each genotype were sacrificed after 2 weeks, 6 months and 12 months, respectively. In a cohort of 15 male WT and *Becn1*^+/−^ mice, cisplatin was injected i.p. (20 mg/kg body weight) after 12 weeks of age. Blood was drawn on day 0 and day 1. Three days after i.p. injection, the mice were sacrificed. To generate primary proximal tubular cells with heterozygous disruption in *Becn1*, *Becn1^+/−^ mice were crossed* to *Tomato/EGFP* reporter-expressing mice. *Gt(ROSA)26Sortm4(ACTB-tdTomato, EGFP)Luo/J* mice were purchased from Jackson Laboratory (Bar Harbour, ME). These mice were crossed to mice with B6;D2-Tg(Slc5a2-cre)1Tauc/Orl (*Sglt2-Cre)* transgene to obtain green fluorescent proximal tubular cells [71]. Sglt2-Cre mice were purchased from EMMA (EM:01098). All the animal studies were approved by the Committee on Research Animal Care Freiburg (G18/08 regional council Freiburg).

### 4.2. Urine and Serum Analysis

Urinary albumin, urinary creatine, serum creatinine and serum urea levels were measured using a fluorimetric albumin test kit (Progen, PR2005, Heidelberg, Germany) and enzymatic colorimetric creatinine and urea kits (Labor+Technik, LT-CR0053, LT-UR0010, Berlin, Germany) following the manufacturer’s specific instructions. Urinary NGAL concentration was assessed in mouse urine after adding 1:1 2-fold Laemmli buffer and boiling for 30 min at 42 °C with subsequent gel electrophoresis and Western blotting. NGAL was detected by using goat anti-NGAL (R&D Systems, AF 1857, Minneapolis, MN, USA ). Samples were normalized for urinary creatinine.

### 4.3. Immunofluorescence Staining

Mouse kidneys were frozen in Tissue-Tek OCT^TM^ (Sakura, SA62550-01, Torrance, CA, USA) and cyrosectioned at 5 μm (Leica Kryostat, Wetzlar, Germany). After fixation with 4% paraformaldehyde, non-specific protein binding was blocked with phosphate-buffered saline (PBS; Thermo Scientific, 10010023, Waltham, MA, USA) containing 5% BSA. Sections were incubated for 1 h with primary antibodies. After washing with PBS, fluorophore-conjugated secondary antibodies (Invitrogen, A-21434, A-11008, A-28180, A-11055, Waltham, MA, USA) were applied for 30 min. The following antibodies were used to evaluate cryosections: goat anti-MEGALIN (Santa Cruz, sc16478, Dallas, TX, USA), fluorescein labeled Lotus Tetragonolobus Lectin (LTG) (VECTOR Laboratories, FL13212, Newark, CA, USA), rabbit anti-LC3B (Cell Signaling 2775, Danvers, MA, USA) and rabbit anti-GFP (Biozol/MBL 598, Eching, Germany).

For paraffin sections, kidneys were fixed in 4% paraformaldehyde and embedded in paraffin. After cutting them into 3 μm sections, they were dehydrated and antigen heat retrieval was performed at a pH of 6. Blocking was performed using PBS containing 5% BSA. Sections were incubated for 1 h with primary antibodies. After washing with PBS, fluorophore-conjugated secondary antibodies (Invitrogen A-21428, A-11055, Waltham, MA, USA) were applied for 30 min. The following antibodies were used for paraffin sections: rabbit anti-cleaved CASPASE 3 (Cell Signaling, 9664, Danvers, MA, USA) and goat anti-MEGALIN (Santa Cruz, sc16478, Dallas, TX, USA). Images were taken using a Zeiss fluorescence microscope equipped with 20× and 63× water immersion objectives.

### 4.4. Histology

Kidneys were fixed in 4% paraformaldehyde, embedded in paraffin and further processed for periodic acid–Schiff (PAS) and hematoxylin and eosin (HE) staining. Quantitative assessment of tubular lesions was performed as previously described via calculation of the percentage of tubules that displayed cell necrosis, loss of the brush border, cast formation and tubular dilatation: Score 0, none; Score 1, ≤10%; Score 2, 11–25%; Score 3, 26–45%; Score 4, 46–75%; Score 5, >76%. At least 10 high-power fields (magnification, ×200) per section for each sample were examined [72].

### 4.5. Cell Lysis and Western Blot Procedures

Cells were harvested by trypsinization, and lysis was performed using RIPA buffer. The protein concentration was determined with a BCA assay (Pierce Biotechnology, 23225, Rockford, IL, USA). Equal amounts of protein were separated on SDS–PAGE gels. Then, the proteins on the gels were transferred to a polyvinyl difluoride membrane (Trans-Blot^®^ Transfer Pack, Bio–Rad 1704157, Hercules, CA, USA) with a semidry blotting technique (Trans-Blot^®^ Turbo™ Transfer System, Bio–Rad 1704150, Hercules, CA, USA). The membranes were blocked in 5% PBS-BSA. The following antibodies were used for Western blotting: rabbit anti-BECLIN 1 (Cell Signaling, 3738S, Danvers, MA, USA), anti-β-ACTIN (Sigma, A5441, Burlington, MA, USA), rabbit anti-LC3B (Cell Signaling, 2775, Danvers, MA, USA), anti-OCT2 (ThermoFisher, ACT-020, Waltham, MA, USA), rabbit anti-GRP78 (Abcam, ab 21685, Cambridge, UK), rabbit anti-CHOP (Cell signaling, 5554, Danvers, MA, USA) and anti-CASPASE 12 (Cell signalling, 2202, Danvers, MA, USA). Densitometry was performed using LabImage 1D software version L320 (Kapelan Bio-Imaging, Leipzig, Germany).

### 4.6. Primary Cell Isolation and Cell Culture

Primary tubular epithelial cells were isolated from 12-day-old *WT;Tomato/EGFP;Sglt2-Cre* and *Becn1^+/−^;Tomato/EGFP;Sglt2-Cre* mice. As recently described, kidneys were dissected and sliced into pieces of 1 mm [73]. After digestion in collagenase, DNAse and proteinase at 37 °C, renal fragments were sieved through two nylon sieves with a pore size of 100 μm and cultured in DMEM-F12 containing 5% FCS, penicillin and streptomycin, epidermal growth factor (EGF), insulin, transferrin, selenit, dexamethason, L-thyroxin and HEPES. After 5 days in culture, renal cells were subjected to FACS to obtain pure primary proximal tubular cells for culturing (GFP-labelled from *Tomato/EGFP x Sglt2-Cre+*). Gene knock-down and overexpression experiments were performed in human embryonic kidney cells (HEK 293T) (purchased at ATCC and cultured in a cell culture incubator at 37 °C, 95% air with 5% CO_2_). Knock-down experiments for *Becn1* were performed by using predesigned pooled siRNA (four targeting one gene, On target SMART pool, Dharmacon, Lafayette, CO, USA). Transfection was performed by using electroporation (Amaxa nucleofector technology, Lonza, Basel, Switzerland) according to the manufacturer’s instructions. Efficiency of the knock-down was confirmed 24 h after transfection using Western blot. For overexpression studies, transient overexpression was performed in HEK cells by using *Becn1* (NM_019584) Mouse Tagged Lenti ORF Clone (Origene Technologies, MR207162L3, Rockville, MD, USA) and electroporation. The efficiency of the overexpression was confirmed 24 h after transfection using Western blot.

### 4.7. Annexin Propidium Iodide Staining

Cells were treated with cisplatin or a vehicle in the concentration indicated. Cells were trypsinized and transferred to annexin binding buffer (cell number 10^6^/mL; pH 7.4,10 mM HEPES; 140 mM NaCl; 2.5 mM CaCl2). Annexin V (Alexa Fluor 647 (ThermoFisher, A23204, Waltham, MA, USA)) was added (final concentration 1%) and cells were incubated for 10 min in the dark. Propidium iodide was added (final concentration 10 µg/mL) and after incubation for another 5 min the fluorescence signal was assessed by using FACSCalibur (Becton Dickinson, Franklin Lakes, NJ, USA).

### 4.8. Statistical Analysis

The data are expressed as the means ± standard deviation (SD). All experiments were performed at least 3 times if not indicated otherwise. Statistical comparisons were performed with 2-tailed Student’s t test with the Excel software program version 14.4.3. Kaplan–Meier-Meier curves were generated and log-rank tests were performed using GraphPad Prism software version 8. Differences with a *p* value ≤ 0.05 were considered to be significant and are marked with *, differences with a *p* value ≤ 0.01 are marked with **, and differences with a *p* value ≤ 0.001 are marked with ***.

## Data Availability

Data are contained within the article and Supplementary Materials.

## Supplementary Materials

The following supporting information can be downloaded at https://www.mdpi.com/article/10.3390/ijms25052560/s1.
