# Supplementary figures and images for: Cisplatin Nephrotoxicity Is Critically Mediated by the Availability of BECLIN1
Source: Int J Mol Sci. 2024 Feb 22;25(5):2560. doi: 10.3390/ijms25052560 (PMC10931997; doi:10.3390/ijms25052560)

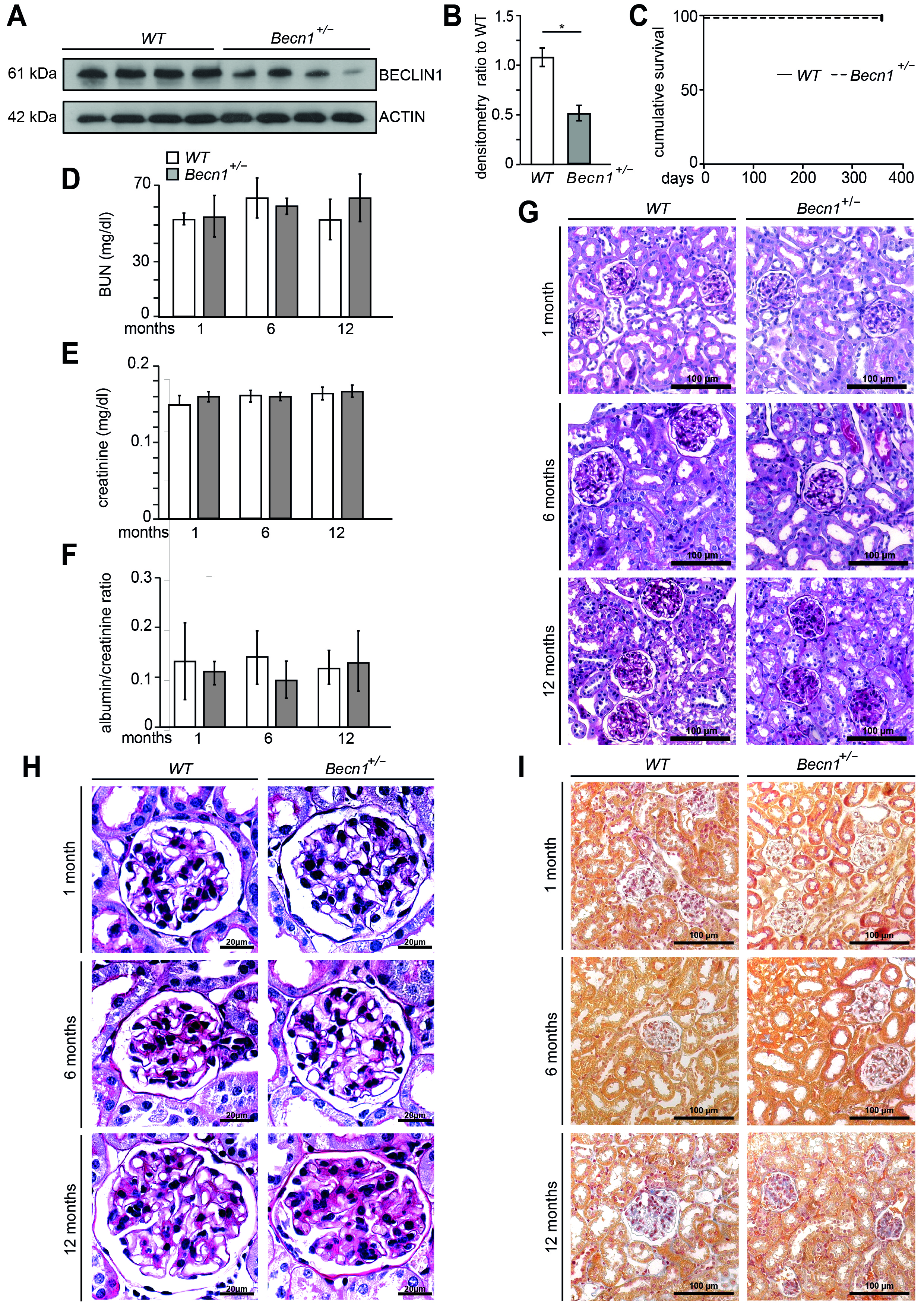

Supplement: Supplementary file 1 [file ijms-25-02560-s001.zip › ijms-2854218-supplementary/Cisplatin_Figure_S1_V10_proof-01.jpg]

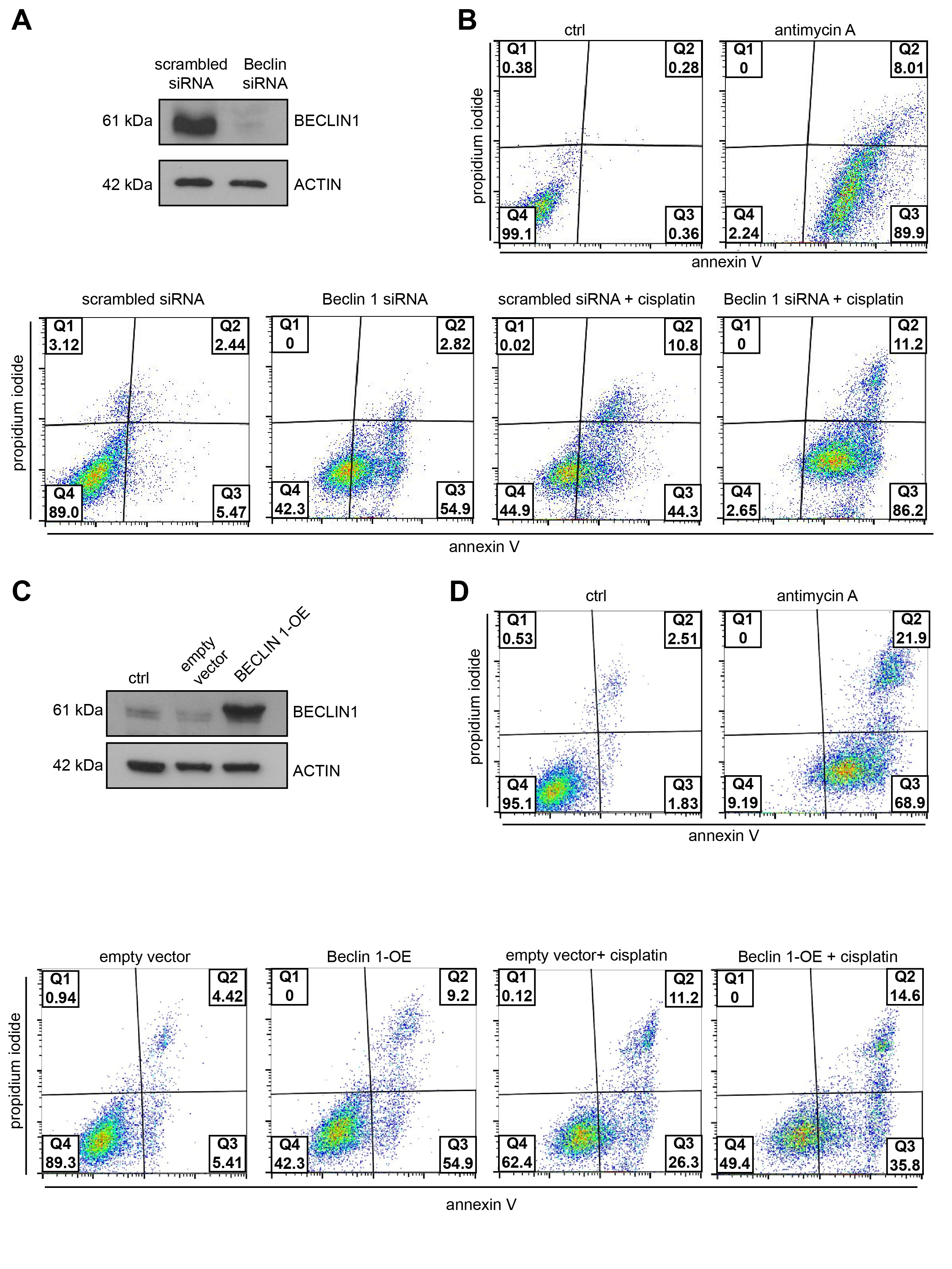

Supplement: Supplementary file 1 [file ijms-25-02560-s001.zip › ijms-2854218-supplementary/Cisplatin_Figure_S2_KD_OE_V10_proof-01.jpg]

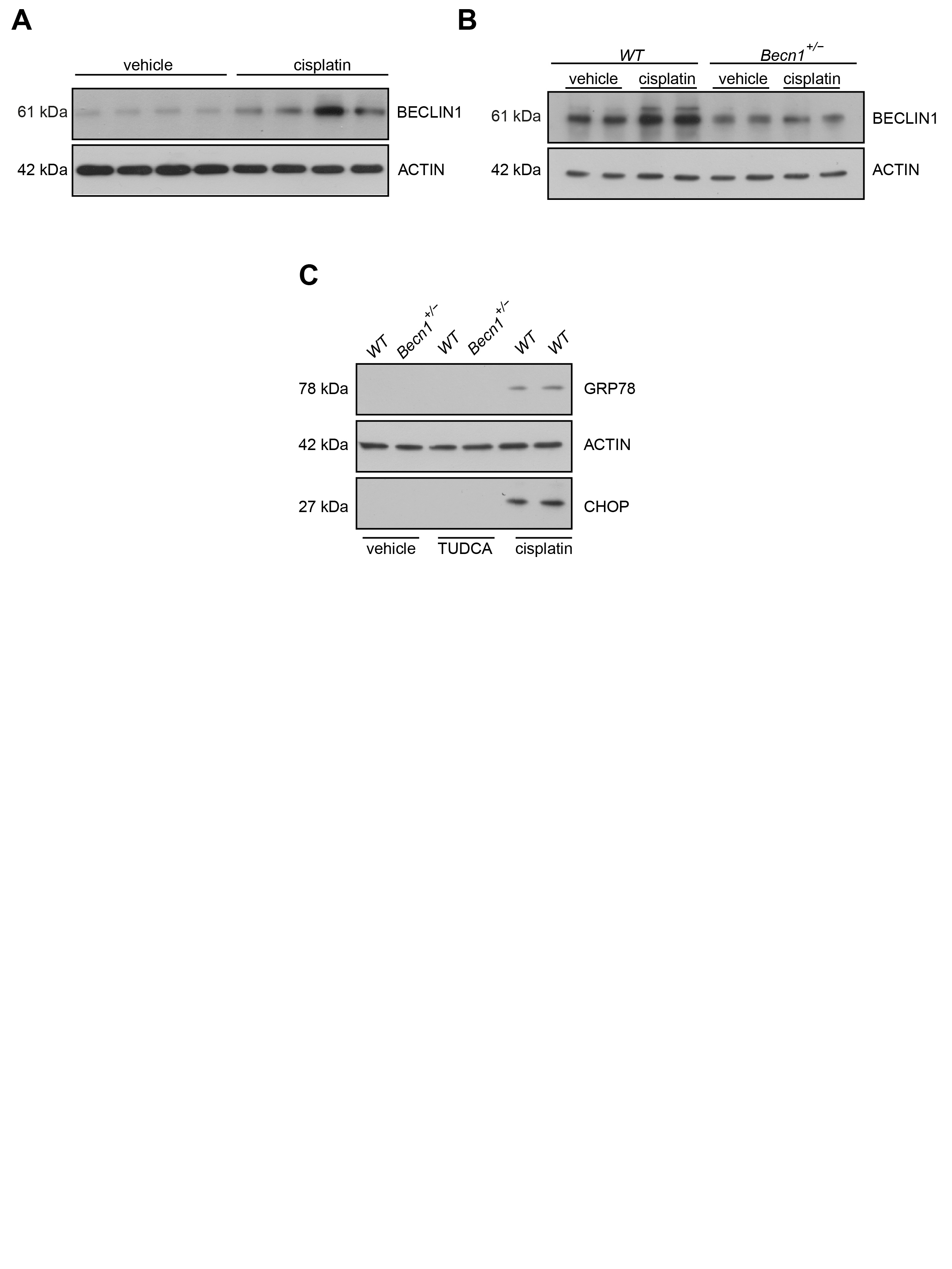

Supplement: Supplementary file 1 [file ijms-25-02560-s001.zip › ijms-2854218-supplementary/Cisplatin_Figure_S3_V10_proof-01.jpg]
